# A pilot randomized controlled trial to assess the effect of Islamic spiritual intervention and of breathing technique with heart rate variability feedback on anxiety, depression and psycho-physiologic coherence in patients after coronary artery bypass surgery

**DOI:** 10.1186/s12991-020-00296-1

**Published:** 2020-08-13

**Authors:** Mohiadin Amjadian, Hadi Bahrami Ehsan, Kaivan Saboni, Siamak Vahedi, Reza Rostami, Daem Roshani

**Affiliations:** 1 Clinical Psychology Department, Kurdistan University of Medical Sciences, Sanandaj, Iran; 2 Psychology Department, University of Tehran, Tehran, Iran; 3 Surgery Department, Medical Sciences University of Kurdistan, Sanandaj, Iran; 4 Cardiology Department, Kurdistan University of Medical Sciences, Sanandaj, Iran; 5 Social Determinants of Health Research Center, Research Institute for Health Development, Kurdistan University of Medical Sciences, Sanandaj, Iran; 6 Department of Epidemiology and Biostatistics, Medical School, Kurdistan University of Medical Sciences, Sanandaj, Iran

**Keywords:** Islamic-religious (spiritual) therapy, Breathing techniques, HRV biofeedback, Depression, Anxiety, Psycho-physiologic coherence

## Abstract

**Background:**

This study investigated the effects of Islamic religious and breathing techniques with heart rate variability (HRV) biofeedback therapies on HRV and psycho-physiologic coherence (resonance frequency), depression and anxiety in coronary artery bypass graft surgery (CABG) patients.

**Methods:**

Sixty CABG patients were chosen and randomly assigned to religious, breathing techniques and control groups. The experimental groups received 8  weeks of treatments; a 2-h session with home works in each week. The control group received only their normal hospital interventions. The groups’ depression, anxiety, HRV and psycho-physiologic coherence levels were assessed before and after the interventions by DASS-21 for depression and anxiety, and em-wave desktop software for HRV and psycho-physiologic coherence. The data were analyzed using ANCOVA with Bonferroni Comparison test and descriptive tests in SPSS software.

**Results:**

The findings showed that there were significant differences in psycho-physiologic coherence (HRV), depression and anxiety scores among the three groups in the post-tests. In fact, depression and anxiety were reduced more in the religious group, while psycho-physiologic coherence raised more in the breathing with the HRV feedback group.

**Conclusion:**

The results showed that both Islamic religious and breathing techniques with HRV biofeedback therapies can be used in rehabilitation programs for CABG patients in clinics and hospitals.

## Background

Cardiovascular diseases are responsible for the highest mortality rate in different parts of the world; they cause 52% and 48% of mortalities in the United States and Europe, respectively [1]. Coronary heart disease (CHD) is one of the major causes of heart failure, and it is the cause of 43% of all deaths in the world [2]. A large number of CHD patients, not treated by medications, undergo coronary artery bypass graft surgery (CABG) [3]; about 60% of all open-heart surgeries are CABGs in Iran [4].

Besides the classic risk factors of the disease (CHD) such as high cholesterol, cigarette smoking, sedentary lifestyle and hypertension, most studies in recent decades showed an association between this disease and psychological disorders such as stress, depression, anxiety and other mood and emotional disorders [5, 6]. Stress, depression and anxiety were common in cardiovascular patients, especially in those undergoing CABG surgeries, and 25% to 50% of these patients were reported to experience such disorders before or after the surgery [7, 8]. Stress, anxiety and depression were also the most important factors affecting quality of life and health status of these patients. In fact, their impact on the patients’ health was reported to be more than left ventricular ejection fraction, angina pectoris, and other chronic diseases as well [9].

Recently, a meta-analysis showed that the patients, having depression after MI, were 2.4 times more likely to be at the risk of death [10]. Glassman and Shapiro, reviewing all the studies published between 1970 and 1998, reported that nine out of ten studies showed a rise in CHD patients’ mortality that was significantly associated with higher prevalence of depression in these patients [11]. In 2012, a meta-analysis on the onset of depression showed that being depressed after an acute heart disease could independently predict mortality and exacerbation of the patients’ heart disease, whether this depression was prior to or after the disease [12]. Depression in patients with CABG was associated with long-term hospitalization, weaker functional outcomes, more pre-operative problems, poor quality of life, more risk of myocardial infarction and higher relapse and mortality rates [13].

Furthermore, anxiety was prevalent in cardiovascular patients and if left untreated, it increased the risk of a subsequent heart attack [14, 15]. Although the prevalence of anxiety in such patients was reported variably in different studies, it was 60% higher on average compared with healthy people [16]; moreover, they experienced higher levels of anxiety compared with other cardiac, cancerous and pulmonary patients [17]. A meta-analysis showed that anxiety was an independent risk factor for heart attack and heart failure in cardiovascular patients [8].

Various studies also reported that 24.7% to 40% of CABG patients underwent depression and anxiety after coronary artery bypass surgery [18, 19]; however, about 65% of these patients was reported to experience such emotional disorders in Iran [20]. Moreover, anxiety seemed to be a disturbing factor in the treatment procedure [21], and the patients undergoing open-heart surgery experienced higher levels of anxiety due to having severe chest pains, fatigue and fear of death, invalidity and aggravating their CHD symptoms despite the surgery [22]. Anxiety also increased postoperative pain, the need for painkillers, pulmonary complications, thrombosis, intestinal dysfunction and risk of infection. In addition, it decreased respiration rate, postoperative vitality, immune response, and caused a considerable delay in postoperative recovery too [23].

## Potential mechanisms

Both behavioral and biological mechanisms were explored as potential pathways linking depression with CVD risk in various studies; in fact, depression was associated with poor adherence to several risk-cutting health behaviors, including physical exercise, smoking cessation, and medications [24, 25]. On the other hand, there was a growing body of evidence linking depression with inflammatory processes-either as a byproduct of these processes or by increasing them [26], dysfunction of autonomic nervous system [27], and impaired coronary blood flow that increased risk of myocardial ischemia [28].

Although the quality of the associations between anxiety or anxiety disorders and CVDs was unclear, some pathways in which anxiety influenced CVD onset or exacerbation were proposed. For example, anxiety was associated with unhealthy behaviors such as cigarette smoking, excessive alcohol intake, lower physical activity [29] and poor diet [30], which increased the risk of CVD.

The most commonly cited biological model proposed a cumulative effect of anxiety on autonomic nervous system activity, which was similar to that proposed for chronic stress and other negative emotions. In that model, anxiety caused excessive activation of the hypothalamic–pituitary–adrenal axis and sympathetic nervous system, and it increased releasing of plasma catecholamine and endothelial damage that finally led to atherosclerosis, coronary artery disease and acute coronary disorders [31].

## Heart rate variability and psycho-physiologic coherence

Heart rate variability (HRV) is a change in the time interval between the heartbeats. Like other body organs, our heartbeat rate is never fixed, and it is always changing. This means that the time interval between heartbeats is either increasing or decreasing, and these changes are the result of the interaction of multiple regulatory mechanisms that affect the heart at different times [32]. On the other hand, depression, anxiety and panics are accompanied by a failure in the autonomic nervous system that partly justifies their risk in such patients.

Moreover, low HRV, hyperactivity of vague nerve, and the sensitivity of the receptors of pressure (baroreflex system) simultaneously with the symptoms of depression were powerful predictors of mortality in CHD patients [33]. Most studies on patients with chronic CHD and those recently underwent acute coronary disorders showed that HRV was lower in depressed and anxious patients compared with the patients with no anxiety and depression [34], and treating depression and anxiety in such patients resulted in their higher HRV and psycho-physiologic coherence.

On the other hand, when certain positive emotional states, such as appreciation, compassion or love were high, heart rhythm patterns were more coherent for longer periods, which also resulted in increasing synchronization among all body systems. In fact, this synchronization-called psycho-physiologic coherence was characterized by distinct, psychological and behavioral as well as specific patterns of physiologic activity in the body systems. Psycho-physiologic coherence (resonance frequency), at the physiologic level, was also characterized by elevating the order, efficiency and coherence of the interactions of body systems such as auto-coherence, synchronization and adaptability [35–37]. This psycho-physiologic coherence was accompanied by an increase in the coherence of cardiac rhythmic activity (cardiac coherence), which represented a pattern of sinusoidal waves at a frequency of about 0.1 Hz. The rhythmic coherence of heart (resonance frequency)—a stable and sinusoidal pattern in the HRV waves—was also the basis of psycho-physiologic coherence, which was characterized by a significant increase in low frequency domain (LF) (particularly about 0.1 Hz), and a decrease in the very low frequency (VLF) and high frequency (HF) ranges.

Moreover, resonance frequency (RF) or rhythmic heart coherence training was one of the primary mechanisms for increasing HRV. Thus, it was possible to determine the specific frequency that stimulates the heart rate to maximize oscillations. Exercise and breathing in specific frequency, relaxation methods and mindfulness brought a kind of stimulation, which maximizes HRV. From the respiratory view, this respiration rate was called resonance frequency, which was between 4 and 7 breaths per minute [38, 39]. Respiratory methods, relaxation, meditation training and the development of positive emotions were ways to increase HRV and RF (40, 41, and 42). In fact, previous controlled trials, which combined slow breathing and providing feedback, reported a rise in the HRV and baroreflex sensitivity (BRS) in patients with CHD [42], chronic pulmonary obstruction [43] and healthy adults [44]. Therefore, the researchers wanted to investigate whether reducing depression and anxiety as negative emotions could increase HRV and psycho-physiologic coherence as physiologic indicators of a healthier heart in CHD patients.

This study investigated possible effects of Islamic religious and breathing technique with HRV feedback therapies on depression, anxiety and psycho-physiologic coherence of CABG patients. In other words, it answered the following questions: Did the religious-based and breathing technique with HRV feedback interventions reduce depression and anxiety of CABG patients? And did they improve psycho-physiologic coherence (resonance frequency) of CABG patients?

## Methods

This was a cross-sectional experimental study with pre-test and post-test conducted in accordance with the Declaration of Helsinki. The inclusion criteria were as follows: Patients with coronary artery bypass graft; age ≤ 70 years; without primary (existing at birth such as developmental and motor skill disorders) and secondary (developing later in life such as substance-induced cognitive impairment) cognitive disorders based on their medical records; and, having finished elementary education-so that the patient himself could complete the required questionnaires. On the other hand, those diagnosed with severe mental disorders such as manic, schizophrenias, etc. were excluded from the study. The sample included all accessible CABG patients, participated in the rehabilitation program in Tohid Hospital in Sanandaj in 2016; in fact, sixty patients, including 19 females and 41 males—between 32 and 67 years—were included in the study by interviewing and studying their medical records. Then, they were randomly assigned to Islamic-religious, breathing techniques with HRV feedback and control groups. No one left the study, and they all took part in the post-tests.

### Assessment tools

DASSS-21 scale for stress, anxiety and depression is a kind of self-reported instrument. Its shortened form has 21 items, which are scored based on a Likert scale as follows; no time = 0, sometimes = 1, most of the times = 2, and almost always = 3. Besharat reported that Cronbach’s alpha coefficients of this scale (*N* = 287) were 0.87, 0.85, 0.89 and 0.91 for depression, anxiety, stress and the whole scale, respectively, in general population samples. These coefficients were also reported to be 0.89, 0.91, 0.87 and 0.93 for depression, anxiety, stress and the whole scale, respectively, in clinical samples (*N* = 194), which confirmed the internal consistency of the scale. Moreover, its concurrent and convergent validities were confirmed through its concurrent administration with Beck Depression and Anxiety Inventory, Positive and Negative Affect inventory, and Mental Health Inventory [45]. However, stress was studied here.

Em-wave desktop software, used for monitoring HRV and psycho-physiologic coherence, was connected to the patient’s body for 5–10 min; then, HR, HRV and a numerical value for the psycho- physiologic coherence or cardiac resonance of the patients were calculated.

## Procedure

The first group, after assessing their anxiety, depression and psycho-physiologic coherence levels, was provided with a religious-based therapy using Islamic and Qur’an teachings followed by doing home works and exercises during the specific hours of days at home—in 2-h sessions for 8 weeks as follows:

- First session: (1) greeting; (2) giving explanations about the concept and aims of the study; (3) determining the timing and duration of the sessions; discussing the lifestyle; (4) talking about spirituality and religion and their impact on mans’ life; (5) explaining the characteristics of spiritual-religious people, and giving exercises.
- Second session: (1) checking exercises of the preceding session; (2) talking about theism, God-oriented life, and the role of belief and trust in God in life; (3) telling religious aphorisms about the impact of trust in God on mental peace; (4) talking about the relation of prayers with peaceful life, and giving exercises (Saying Dhikr & prayer for himself and others).
- Third session: (1) reviewing the main agenda of the preceding session; (2) talking about the role of reliance and trust in God in life for improving the spiritual health; (3) proposing verses and narratives, and specifying examples in the participants’ own life and giving exercises (Saying Dhikr-La hawla wa la ghowta ela bellah & prayer for himself and others).
- Fourth session: (1) reviewing the main agenda of the preceding session; (2) discussing the role of thanking God; (3) defining appreciation and thanking and proposing several Hadiths and narratives in this regard, and giving exercise (Saying the Dhikr–Alhamdolelah, and prayer for himself and others).
- Fifth session: (1) reviewing the main agenda of the preceding session; (2) being familiar with forgiveness and discussing the key role of forgiveness in improving the spiritual health; (3) proposing Ahadith and narratives about the role of forgiveness in life; (4) Pointing out the consequences of participating in charity affairs, and giving exercise (planning to visit and help the invalids in a nursing house this week).
- Sixth session: (1) reviewing the main agenda of the preceding session; (2) explaining the role of Dhikr, prayers, supplications, and pilgrimage in improving the spiritual health and its effects on personal life; (3) practicing them with the patients, and giving exercises (Saying Dhikrs and prayer for himself and others).
- Seventh session: (1) reviewing the main agenda of the preceding session; (2) referring to the role of patience in life and improving the spiritual and mental health, and giving exercise (Listening to Ahadith and narratives about the role patience in life, especially when someone is ill).
- Eighth session: (1) talking about how to release emotions and feelings; (2) talking about forgiveness, acknowledging and praying the creator of the universe; (3) reviewing the program and aims, evaluating the stated subjects, getting feedbacks from the participants, questions and answers, concluding the whole program, and completing the questionnaires again, finishing the session.

The second group, after pre-tests, was individually trained deep and slow breathing techniques. In the first session, each patient’s baseline psycho-physiologic coherence number was calculated by em-wave desktop software. Then, the patients were individually attached to the HRV and psycho-physiologic coherence monitoring through a computer, and they were simultaneously taught how to harmonize their breathing to achieve higher levels of psycho-physiologic coherence in 2-h weekly sessions for 8 weeks. The patients received deep and slow breathing training and HRV feedback to achieve their optimal psycho-physiologic coherence. Moreover, they were asked to practice the techniques based on a timetable during the week, especially before going to bed. At the end of the eighth week, the two groups’ anxiety, depression, HRV and psycho-physiologic coherence levels were reassessed.

### Statistical analysis

The Analysis of Covariance Method (ANCOVA) with Bonferroni Comparison test was used to analyze the data. Moreover, Shapiro- Wilkes and Leven tests were used to check the normal distribution of data, error terms, and to test the homogeneity variance of the dependent variables and error variances. *P* value less than 0.05 was considered statistically significant for any test.

## Results

Table 1 shows that, out of 60 patients, 71% were male, and their mean age was 55.67 years. They participated in the study 6–12 weeks after their bypass surgery. In addition, 70% (42 patients) had AMI or minor strokes before CABG, and 52% had three; 46% had two, and about 2% had four new routes in their CABGs. Moreover, about 35% of the patients underwent some kind of arrhythmia before CABG, and none of the patients reported alcohol or illicit substance abuse. The characteristics of the sample are summarized in Table 1.

**Table 1:** Mean ± standard deviation on baseline DASS-21, spiritual health and HRV scores and some characteristics of the cardiovascular patients

	Islamic Intervention Group	Breathing Intervention Group	Control Group
Baseline Psycho-physiologic Coherence (HRV)	0.375 ± 0.148	0.448 ± 0.244	0.47 ± 0.273
Baseline anxiety	17 ± 6.14	12.7 ± 7.05	14.8 ± 5.61
Baseline depression	19.4 ± 9.262	17.2 ± 10.923	14.4 ± 7.358
Baseline spiritual health	86.873 ± 7.97	79.481 ± 17.506	89.584 ± 6.101
Age	55.8 ± 7.19	56 ± 9.51	59.55 ± 7.2
Number of weeks elapsed since CABG	9.35 ± 1.42	9.15 ± 1.53	9.05 ± 1.43
Sex(Female)	7 (35%)	5 (25%)	6 (30%)
AMI before CABG (chronic failure)	14 (70%)	13 (65%)	14 (70%)
Number of affected coronary vessels			
Two vessels	8 (40%)	9 (45%)	11 (55%)
Three vessels	12 (60%)	11 (55%)	8 (40%)
More than three vessels	0	0	1 (5%)

The data showed that the baseline mean scores for psycho-physiologic coherence were 0.375, 0.448 and 0.47 in religious, breathing and control groups, respectively (The scale was scored between zero − 1 with three different levels; 0.00–0.35, 0.35-0.7 and 0.7–1 which were considered as low, medium and high levels of psycho-physiologic coherence). Having the ANCOVA assumptions confirmed, covariance analysis of psycho-physiologic coherence in the three groups showed that its difference among the three groups, while the effect of the confounding factors controlled, was significant (*F* = 14.193, *P* = 0.000). The corrected squared R showed that about 31% of the variation of this variable was due to the effects of the therapies. In other words, the interventions could change it significantly in the experimental groups (0.8 and 0.911 in post-test), while there was no significant change in the control group (0.335 in post-test) (Table 2).

**Table 2:** Tests of between-subjects effects for psychophysiological coherence before intervention

Source	Type III sum of squares	*df*	Mean square	*F*	*P* value	Partial eta squared^a^
Group	6.714	2	3.357	14.193	0.000	0.336
Psychophysiological Coherence	0.578	1	0.578	2.445	0.124	0.042
Error	13.245	56	0.237			
Total	56.91	60				

^a^*R* Squared = 0.346 (Adjusted *R* Squared = 0.311)

Moreover, multiple comparisons using Bonferroni test showed that there was a difference in increasing the patients’ psycho-physiologic coherence in the three groups (Table 5). The mean differences in the groups indicated that the difference between the breathing and the control groups (0.576), and the religious and the control groups (0.465) were significant at *P* < 0.05. There was also a difference between the two experimental groups (0.111), indicating a further increase in the patients’ psycho-physiologic coherence in the breathing techniques with HRV group.

On the other hand, the baseline depression scores for the religious, breathing and control groups were 19.40, 17.20 and 14.40, respectively. The analysis of covariance of depression in the three groups showed that its difference among the groups, while the effect of the convergent variable controlled, was significant (*F* = 12.552, *P* = 0.000). The corrected squared R showed that about 34.5% of the variation of this variable was because of the effects of the therapies. In other words, the interventions were able to change the patients’ depression in the experimental groups significantly, but no significant change happened in the control group’s pre and post-test scores, 14.4 vs. 15.26 (Table 3).

**Table 3:** Tests of between-subjects effects for depression before intervention

Source	Type III sum of squares	*df*	Mean square	*F*	*P* value	Partial eta squared^a^
Group	897.751	2	448.876	12.552	0.000	0.31
Depression	524.953	1	524.953	14.679	0.000	0.208
Error	2002.647	56	35.762			
Total	8752	60				

^a^*R* Squared = 0.379 (Adjusted *R* Squared = 0.345)

Furthermore, multiple comparisons by Bonferroni test showed that there was a significant difference in reducing the patients’ depression in the three groups (Table 5). The mean differences among the groups showed that the differences between the breathing and control groups (8.318), and the religious and control groups (8.360) were significant at *P* < 0.05. There was also a difference between the two experimental groups (0.042) indicating more reduction in depression scores in the religious group.

The data also showed that baseline anxiety scores for the religious, breathing and control groups were 17, 12.7 and 14.8, respectively. The analysis of covariance of anxiety in the three groups showed that its difference among the groups, while the effect of the confounding factors controlled, was significant (*F* = 7.337, *P* = 0.001). The corrected of squared R showed that 17.1% of the variation of this variable was due to the effects of the therapies. In other words, the interventions were able to change the patients’ anxiety significantly in the experimental groups, but no significant change happened in the control group’s mean scores in pre-test and post-test (14.80 vs. 10.50) (Table 4).

**Table 4:** Tests of between-subjects effects for anxiety before intervention

Source	Type III sum of squares	*df*	Mean square	*F*	*P* value	Partial eta squared^a^
Group	370.079	2	185.04	7.337	0.001	0.208
Anxiety	56.124	1	56.124	2.225	0.141	0.038
Error	1412.276	56	25.219			
Total	4848	60				

^a^*R* Squared = 0.213 (Adjusted *R* Squared = 0.171)

Besides, multiple comparisons by Bonferroni test also showed that there was a significant difference in reducing anxiety in the three groups (Table 5). The mean differences among the groups showed that the differences between the breathing and control groups (6.10), and the religious and control groups (4.21) were significant at *P* < 0.05. There was also a difference between the two experimental groups (1.89) indicating more reduction in anxiety scores in the breathing group.

**Table 5:** Result of Bonferroni comparison test

Dependent variable	(*I*)Group	(*J*)Group	Mean difference (*I* − *J*)	Std. error	*P* value	95% confidence interval	
						Lower bound	Upper bound
Coherence	Religious	Breathing	− 0.111	0.155	0.349	− 0.494	0.171
	Religious	Control	0.465^a^	0.155	0.002	0.111	0.88
	Control	Breathing	0.576^a^	0.155	0.000	0.18	0.95
Depression	Religious	Breathing	− 0.042	2.1	0.9	− 5.8	4.6
	Religious	Control	8.36^a^	2.1	0.005	3.1	13.5
	Control	Breathing	8.318^a^	2.1	0.002	3	13.3
Anxiety	Religious	Breathing	− 1.89	1.6	0.375	− 4.46	0.77
	Religious	Control	4.21^a^	1.6	0.153	1.76	8.26
	Control	Breathing	6.10^a^	1.6	0.002	2.14	10.06

^a^The mean difference is significant at 0.05 level

In general, the results suggested that the patients in religious and breathing groups, compared with the patients in the control group, showed a significant reduction in their anxiety and depression, and a significant increase in their psycho-physiologic coherence scores.

## Discussion

This study investigated the effects of religious and breathing techniques with HRV biofeedback therapies on psycho-physiologic coherence, depression and anxiety of CABG patients. The findings showed that there was a significant inverse correlation between the patients’ heart rate variability (psycho-physiologic coherence) and their depression and anxiety. These results were in line with the findings of the studies that reported the positive effect of spirituality and religious activities on reducing psychological disorders such as depression, anxiety and stress in such patients [44, 46–51].

Moreover, the findings were in line with the results of Sahraian et al. that reported spirituality and religious activities as important social resources of reducing mental disorders such as depression and anxiety, and of coping with chronic diseases [52]. Increasing spiritual well-being by engaging the patients in religious activities was also associated with faster recovery, increased efficacy and general health, and it was also reported to reduce cardiovascular events, blood pressure, Alzheimer’s disease, diabetes and cancer risks [53–55] which were in line with the findings of this study.

Several pathways were suggested to show how religion and religious activities affect cardiovascular health as follows [56]:

1. Psychological pathway: Spirituality and religion provided good psychological resources to match and cope with the problems by: (a) reducing the likelihood of stressful events in life (due to change in behavior); (b) giving meaning and purpose to stressful events in life; and (c) providing social symbols such as resilient individuals who lived in misery and faced many problems, but they were spiritually strong. This led to experiencing more positive emotions (Optimism, hope, etc.) and fewer negative feelings (depression, anxiety, frustration, etc.), which had positive effects on the immune, endocrine, nervous and inflammatory systems, leading to healthier cardiovascular system in such patients.
2. Social pathway: Spirituality and religion were good resources for coping and adapting patients to stressful situations, and they acted as a social buffer against negative emotions by integrating them in spiritual and religious communities, which help them internalize forgiveness and other good social views. These social and friendly gatherings also provided more opportunities for health information exchange for those participating, and provided them with more healthcare resources. In this study, the patients were engaged in spiritual and religious activities to benefit from these social advantages.
3. Behavioral pathway: Spirituality and religion promoted overall health and reduced the incidence of cardiovascular diseases in particular, by providing specific behavioral plans in various religious contexts (e.g., Christian and Islamic religious health orders) such as smoking cessation advice, prohibiting free sexual intercourse, drug abuse and alcohol intake, and encouraging people to have special diets such as fasting, to do exercise and to involve in certain social activities. They, on the other hand, encouraged people to refrain from lying, to be responsible and honest, to increase their adherence to the medical orders, which helped them recover from cardiovascular problems sooner.
4. Supernatural pathway: Some scholars attributed positive association between religion/spirituality and general and cardiovascular health to a supernatural source such as God. The classic examples were the double-blind studies in which some ones prayed for someone else (CABG patients), such as Bird’s (1988) study at San Francisco General Hospital, the study of the American Heart Institute in Kansas, the study of Harris et al., and the multi- dimensional studies at Harvard by Benson et al. [57–59].

The findings also showed a significant inverse correlation between the patients’ HRV and their depression and anxiety; in fact, breathing techniques with HRV feedback was significantly effective on reducing anxiety and depression, and increasing psycho-physiologic coherence of the patients, which were in line with the findings by Nolan et al.; Del Pezo et al.; Giardino, Chan and Borson; Lehrer et al.; Lehrer, Vaschillo and Vaschillo; Vaschillo et al. They reported that breathing techniques with HRV feedback was significantly effective on increasing the patients’ psycho-physiologic coherence and their physical and mental health, and decreasing their depression and anxiety as well [36, 38, 40, 41].

Steven Porges suggested that the main component of respiratory sinus arrhythmia (RSA) was the activity of the vagus nerve [60, 61]. The vagus nerve is the tenth nerve of 12 pairs of cranial nerves. Its parasympathetic nerves are branched into different body organs, such as heart, lungs and stomach. Porges proposed poly-vagal theory; this theory considered the role of the vagus nerve from an evolutionary perspective. According to this theory, the autonomic nervous system of man has been evolved with three distinct circuits: immobility, mobility, and social communication or behavior. Porges claimed that non-stimulation or stimulation of the vagus nerve to the heart could cause arousal or calmness in a person. That is, no activity in the vagus nerve stimulated the autonomic nervous system, whereas the vagus nerve stimulation caused it to turn off. Porges suggested that RSA reflected the parasympathetic or vagus nerve’s activity. The vagus nerve’s activity acted as a brake that could slow down the heart rate during an exhalation, while prohibiting its activity could increase heart rate during inhalation.

On the other hand, strong vagal rhythm was important for proper body autonomous function such as acceptable RSA and HRV activities. Strong vagal rhythm was particularly essential for maximizing heart rate during inhalation and minimizing it during exhalation [62]. Another advantage of RSA was to increase pulmonary function. As described by Yasuma and Giardino, RSA promoted respiratory function by increasing blood flow during inhalation, and while oxygen concentration was the highest in the alveolus [41, 62].

The second source of HRV was Baroreflex system activity. Paul Lehrer provided a comprehensive description of the role of this system on the HRV level [36]. Baroreflex was the body’s ability to regulate blood pressure. Pressure receptors (baro-receptors) were tensile receptors located in the Aorta and Carotid arteries (SATs). They reacted to changes in the diameter of these blood vessels. Pressure receptors, in response to hypertension, sent a signal to decrease heart rate and vascular resistance (increased blood vessel diameter), which ultimately led to lowering blood pressure. When pressure receptors recorded blood vessels’ dilatation and decreased blood pressure, they sent signals to the brain to increase heart rate and vessel contraction, and this cycle continued. Therefore, Baroreflex was a negative feedback mechanism that helped maintain body homeostasis.

The strength and power of Baroreflex were measured through units of change in pulse rate or beats on ECG (in milliseconds) per unit of change in blood pressure (in mmHg, Mercury millimeters). Given that HRV was the time variation between the beats, the Baroreflex and HRV ratios were clear—the stronger Baroreflex was, the higher HRV level and vice versa [36, 63]. Therefore, breathing techniques increased HRV and psycho- physiologic coherence through the respiratory sinus arrhythmia and Baroreflex systems that seemed to decrease anxiety and depression and improve cardiovascular health in these patients.

This study was innovative, because spiritual interventions used in earlier studies were mostly based on eastern meditation or spirituality based on Christianity, while the religious intervention in this study, was designed and carried out based on the teachings of Islam and cultural and religious backgrounds of the patients’ social life. In addition, it was one of the first studies in Iran, which used HRV index and religious intervention simultaneously, and monitored the patients’ cardiac health through checking their psycho-physiologic coherence levels. Moreover, this study showed that Islamic religious intervention could reduce depression and anxiety of CABG patients by increasing their HRV and psycho- physiologic coherence, which could be monitored directly by the researchers.

## Conclusion

The findings showed that the religious intervention based on Islamic and Quran teachings could significantly decrease depression and anxiety, and it also increased psycho-physiologic coherence and HRV of CABG patients. Since increasing HRV improved the patients’ cardiovascular and general health, it is recommended to carry out more studies in different groups to shed further lights on the effects and outcomes of this intervention, if it is to be used in cardiac rehabilitation programs for such patients in the future.

## Limitation

This study had the following limitations: (1) lack of follow-up; (2) not checking the patients’ religious background and views after the experiments; (3) a small sample size; (4) not being able to check the fidelity and adherence of the experimental groups doing their daily home works and exercises; (5) the findings might not be generalizable to other communities with different religious backgrounds; (6) the sample was heavily male (perhaps due to the higher number of CHD patients among men); (7) this was a “no-treatment” control trial, i.e., the “control group” received their regular medical treatment just as did the other two groups (no placebo treatment for any group).

Suggestions: It is suggested to use Islamic religious intervention with bigger samples of CHD patients to achieve more valid and reliable findings, if we are going to use it for such patients in the future. It is also recommended to repeat the study on the patients with various religious backgrounds and physical problems such as cancerous, diabetics, etc. Finally, it is suggested to design and conduct follow-up studies with such patients to investigate possible effects and outcomes of this intervention in longer times.

## Data Availability

The data used to support the findings of this study are available from the corresponding author upon reasonable request.
